# The Co-existence of ADHD With Autism in Saudi Children: An Analysis Using Next-Generation DNA Sequencing

**DOI:** 10.3389/fgene.2020.548559

**Published:** 2020-12-15

**Authors:** Neda M. Bogari, Faisal A. Al-Allaf, Ashwag Aljohani, Mohiuddin M. Taher, Nermeen A. Qutub, Suhair Alhelfawi, Amal Alobaidi, Derar M. Alqudah, Hussain Banni, Ghida Dairi, Amr A. Amin

**Affiliations:** ^1^Department of Medical Genetics, Faculty of Medicine, Umm Al-Qura University, Makkah, Saudi Arabia; ^2^Science and Technology Unit, Umm Al-Qura University, Makkah, Saudi Arabia; ^3^Special Need Department, School of Education, Umm Al-Qura University, Makkah, Saudi Arabia; ^4^Institute of Education, University of Reading, Reading, United Kingdom; ^5^Sinad City for Special Education, Jeddah, Saudi Arabia; ^6^Medicine and Medical Sciences Research Center, Deanship of Scientific Research, Umm Al-Qura University, Makkah, Saudi Arabia; ^7^Department of Physiology, College of Medicine, King Saud University, Riyadh, Saudi Arabia; ^8^Department of Biochemistry, Faculty of Medicine, Umm Al-Qura University, Makkah, Saudi Arabia; ^9^Faculty of Medicine, Ain Shams University, Cairo, Egypt

**Keywords:** ADHD (attention deficit and hyperactivity disorder), autism (ASD), GWAS, SNP, learning disorders

## Abstract

Attention-deficit/hyperactivity disorder (ADHD) is one of the most common neurodevelopmental disorders. Several studies have confirmed the co-existence of other neuropsychiatric disorders with ADHD. Out of 106 individuals suspected to have ADHD, eight Saudi Arabian pediatric patients were diagnosed with ADHD using a dual assessment procedure based on highly significant scores from the international criteria for diagnosis; (full form DMS) DSM-5. Then, these patients were examined for the co-existence of autism and ADHD using different international diagnostic protocols. Four patients with combined ADHD and autism and four ADHD patients without autism were examined for the presence of genetic variants. Six variants (chr1:98165091, chr6:32029183, chr6:32035603, chr6:32064098, chr8:2909992, chr16:84213434) were identified in 75% of the patients with ADHD and autism, indicating that these genes may have a possible role in causing autism. Five variants (The chr2:116525960, chr15:68624396, chr15:91452595, chr15:92647645, and chr16:82673047) may increase to the severity of ADHD. This study recommends screening these eleven variants in ADHD cases and their relevant controls to confirm the prevalence in the Saudi population. It is recommended that future studies examine the 11 variants in detail.

## Introduction

Attention-deficit/hyperactivity disorder (ADHD) is one of the most widespread neurobehavioral disorders presenting for treatment in children (American Academy of Pediatrics, Subcommittee on Attention-Deficit/Hyperactivity Disorder Committee on Quality Improvement, 2001). According to a previous study that assessed the ADHD prevalence in ten countries in the Americas, Europe and the Middle East, the estimates prevalence was 3.4% (Fayyad et al., 2007). However, a recent meta-analysis study manifested that variations in the characteristics of the studies have a huge impact on the variations of the ADHD prevalence rates (Polanczyk et al., 2014). Indeed, behavioral therapy and medications are a key treatment for this disorder. However, evidence from literature recommended a conducting of more research interventions to understand and optimize treatment effectiveness (Daley et al., 2018). While the features of attention-deficit hyperactivity disorder (ADHD) are characterized by inattention and hyperactivity-impulsivity, studies have shown that about one fifth of the children and adolescents with ADHD meet the diagnostic criteria of ASD (Hollingdale et al., 2020). In review, two-thirds of individuals diagnosed with ADHD exhibit characteristics of autism spectrum disorder (ASD), which is characterized by core social communication impairments and restrictive-repetitive behaviors (Davis and Kollins, 2012). Although the co-occurrence of ADHD and ASD was recognized decades ago, research focusing on co-occurring ADHD and ASD has only recently emerged (Rommelse et al., 2010). Currently, the Diagnostic and Statistical Manual of Mental Disorders (DSM-V) allows, for the first time, the diagnosis of the co-occurence of both disorders. There is an evidence that when ADHD co-exists with ASD, the impact can be critically severe on an individual’s life (Yerys et al., 2009). A study conducted by Rao and Landa (2013) compared affected individuals with both ADHD and ASD to individuals with a single diagnosis (Ding et al., 2013). The results show that the co-occurrence may increase the severity level of deficiency, and it may decrease the benefit of the delivered treatments and interventions. Furthermore, studies have suggested that the frequent co-existence of ADHD and ASD increases the likelihood of the manifestation of other disorders, such as bipolar disorder, sleep problems, and obsessive-compulsive disorder, which lead to severe impacts on an individual’s life quality (Mariani and Levin, 2007).

This study aimed to evaluate the co-existence of autism (clinically and genetically) with ADHD in children using whole-exome sequencing, and to identify the possible associated risk-variants for this co-existence, describing the underlying genetic causes of autism developing among ADHD infected-children using advanced filtration and predicting programs. We suggest that genetic approaches including whole exome sequencing related-studies may help in developing new treatment modalities and better understanding the mechanisms underlying the co-existence of these disorders.

## Materials and Methods

### Study Design and Clinical Presentation

This study was approved by the Medical Ethical Committee of the Faculty of Medicine, Umm Al-Qura University (ethical approval no. HAPO-02-K-012-2017-01-204) and was conducted in accordance with the principles of the Declaration of Helsinki. An electronic observation questionnaire was constructed to survey ADHD symptoms and was distributed to all available centers, schools, and hospitals. The total number of individuals who responded was 106, with an age range of 6–14 years old and 58 males and 48 females. Most of the electronic responders were already diagnosed either nationally (in Saudi Arabia) or abroad. Eight Saudi Arabian pediatric patients were determined to have ADHD after clinical reassessment [i.e., they met the criteria used by the study with significant scores on the Attention-Deficit Hyperactivity Disorder Clinical List, which was derived from the Diagnostic and Statistical Manual of Mental Disorders – fifth edition (DSM-5) (Davis and Kollins, 2012)]. Four of the patients with ADHD were diagnosed with autism associated with ADHD (combined group). Consent to participate in this study was obtained from the legal guardians of all patients.

The study designed a protocol to diagnose attention-deficit and hyperactivity disorder that was derived from the Diagnostic and Statistical Manual of Mental Disorders – fifth edition (DSM-5) criteria and included a list of 9 parameters for attention deficit and 9 parameters for hyperactivity and impulsivity.

The list was designed as a 3-point scale as follows (parameter does not exist = 0, parameter is partly present = 0.5, parameter is fully present = 1). The study protocol showed high psychometric characteristics, based on both the participants’ responses to a question on a 3-point frequency scale and values of the Pearson correlation coefficient as a measure of internal consistency.

Validity was calculated between the participants’ scores on each dimension and the total score of the scale, as shown in table (Davis and Kollins, 2012).

Regarding the autism assessment protocol, the study used four different protocols. The first protocol was the test designed by the Stanford-Binet Intelligence Scales – 5th edition, which measures five cognitive abilities: knowledge, working memory, quantitative reasoning, fluid reasoning, and visual-spatial processing in two domains (verbal and non-verbal). The test provides a full-scale IQ that describes an individual’s cognitive ability with average score of 90–119 (Roid, 2003).

The second protocol used for the autism clinical assessment was based on the Vineland-II Adaptive Behavior Scales (VABS), which assesses the adaptive functioning of individuals from birth to 18 years old. It involves four major domains (communication, socialization, daily living skills, and motor skills) that form the final composite score.

The third protocol was the Gilliam Autism Rating Scale – Second Edition (GARS-2), a norm-referenced screening instrument used to assess the severity of ASD in individuals aged 3–22 years. The assessment contains 42 items grouped into three subtests (stereotyped behavior, communication, and social interaction) that describe the characteristics of ASD (Gilliam, 2006). The fourth protocol, the Autism Behavior Checklist (ABC), is a checklist that was developed for screening the severity of autism in affected individuals. It consists of 57 items that reflect an individual’s challenges in four areas: sensory behaviors, language behaviors, body and object use behaviors, and relating behaviors (Rellini et al., 2004). After assessment, as mentioned in Table 1, patients 1–4 had both ADHD and autism and patients 5–8 had only ADHD.

**TABLE 1:** Summary of selected patients’ phenotypes and characteristics.

**ADHD Patient No.**	**Age at Diagnosis (years)**	**Sex**	**Results of Attention**	**Results of Hyperactivity**	**Total**
1 (+Autism)	6	M	8.5	7	15.5
2 (+Autism)	8	M	7.5	7.5	15
3 (+Autism)	9	M	9	6	15
4 (+Autism)	6	M	8.5	6.5	15
5	11	F	9	9	18
6	6	M	7.5	9	16.5
7	9	F	9	7.5	16.5
8	11	M	7	8	15

### DNA Isolation and Whole-Exome Sequencing

Genomic DNA was extracted from EDTA-anticoagulated peripheral venous blood samples using the PureLink Genomic DNA Mini Kit from Applied Biosystems (catalog number: K182001). Genomic DNA was quantified using the Qubit-dsDNA HS (High Sensitivity) Assay Kit (Thermo Scientific). Whole-exome sequencing was performed for patient DNA samples using the Ion Torrent technology from Thermo Scientific. Genomic DNA (50–100 ng) was subjected to enriched library preparation with an Ion AmpliSeq Library Kit Plus kit (Cat. No. 4488990), with a mean amplicon size of 215?bp, and with an ion AmpliSeqTM Exome RDY Kit 1 × 8 containing primer pools (Cat. No. 4489838, Life Technologies) following the manufacturer’s protocol then quantified using qPCR with an Ion Library TaqMan quantitation kit (Cat. No. 4468802). Each sample was assigned a distinct barcode using the IonXpress barcode kit (Cat. No. 4471250). Barcoded libraries were diluted to 100 pmol, and sample libraries were pooled and subjected to template preparation on ion sphere particles (ISPs) using the Ion OneTouch 2 System and the Ion PI HI-Q Template OT2 200 kit (Cat. No. A26434). Each templated ISP was loaded on an Ion PI v3 chip (Cat. No. A26770) and sequenced on an Ion Proton instrument (Life Technologies, United States) using an Ion PI Hi-Q Sequencing Kit (Cat. No. A26772). The alignments were performed by Torrent Suite v4.2. Filtering low-quality reads, adapter removal followed alignment against the human reference genome (hg19 build). Bioinformatic analysis was performed using Torrent Suite Software (v5.10.1.0), and the variants were called using the Torrent Variant Caller plugin (v5.10) and then imported into the Ion Reporter software (v5.10) for the annotation. Gene variants located within exons, introns, predictive splice sites and UTRs were identified with less than 1% minor allele frequency (MAF). Functional exons variants were also identified as frameshift, synonymous, missense, nonsense and stop loss variants.

### Biostatistical Analysis and Filtration

A total of 271 genes were selected from the ADHD genome-wide association study (GWAS) database (according to genes listed in the GWAS catalog on 3/7/2018). However, only 252 genes were used in filtration in this study because 19 of the genes did not match the Ion Torrent library kit. A total of 531 genes were selected from the autism genome-wide association study (GWAS) database (according to genes listed in the GWAS catalog on 3/7/2018). However, only 379 genes were used in filtration in this study because 131 of the genes did not match the Ion Torrent library.

All samples selected (the ADHD non-autistic group and the ADHD autistic group) were uploaded into Ion Reporter v5.6, where exonic nonsense, missense, frameshift insertion, and frameshift deletion variants were included, and all resulting gene variants were annotated and filtered for functional consequences using Ion Reporter software. Ninety two genes were present in both groups (ADHD alone and ADHD co-existing with autism).

Four algorithm tests for predicting the health risk burden at the population level were used while introducing huge sequencing data-results to predict whether this SNP variants benign or harm. The tests included the multi-step procedure; Sort Intolerant From Tolerant (SIFT) which searches for sequences similarities, calculating probabilities of all possible substitutions at different position of alignments (Ng and Henikoff, 2003), PhyloP score which evaluate the signatures of selection at particular nucleotides by measuring evolutionary conservation; Grantham score which predict the evolutionary distance between two amino acids based on the chemical properties predicting the effect of substitutions between two amino acids; PolyPhen-2 score which predicts the probability of the effect of a substituted amino acid on the structure and function of the protein (Ng and Henikoff, 2001).

## Results

Table 2 and Supplementary Table S1) show the filtered variants identified for ADHD genes in the eight patients (patients 1–8). Filters were applied in order to examine pathogenic variants. The filters include exonic variants, a sorting intolerant from tolerant (SIFT) score < 0.05 and a PolyPhen score > 0.5. Following filtration, one missense variant (chr13:29898768) was found in all patients, and another missense variant (chr17:3657159) was found in seven out of the eight patients. Two missense variants (chr12:89916811, chr16:81249954) were identified in six patients, and three other missense variants (chr2:133541107, chr11:63974966, chr12:89745477) were found in four patients. Six missense variants (chr6:12120588, chr8:2909992, chr8:72977703, chr16:81241100, chr16:81249927, chr17:3632836) were found in five patients. Another six missense variants (chr2:133540605, chr2:133542585, chr9:1056959, chr12:100042040, chr12:106460938, chr16:81211496) were identified in only three of the eight patients.

**TABLE 2:** Rare and novel variants of ADHD genes identified by exome sequencing in the 8 Saudi Arabian ADHD patients.

**Locus**	**Ref**	**Genes**	**rs ID number**	**Samples**								**Amino Acid Change**	**Coding**
				**1**	**2**	**3**	**4**	**5**	**6**	**7**	**8**		
chr1:70505307	A	LRRC7	Novel			A/G						p.Glu1229Gly	c.3686A>G
chr1:70541953	A	LRRC7	Novel			A/C						p.Gln1437Pro	c.4310A>C
chr1:200968575	T	KIF21B	rs753390486								T/G	p.Glu596Ala	c.1787A> C
chr1:218520315	G	TGFB2	rs10482721		G/A							p.Arg91His	c.272G>A
chr2:111598958	C	ACOXL	rs1554005								C/T	p.Thr255Met	c.764C>T
chr2:111850515	C	ACOXL	rs17041850						C/T			p.Pro505Leu	c.1514C>T
chr2:133489376	C	NCKAP5	rs776162189						C/G			p.Asp1793His	c.5377G>C
chr2:133538651	C	NCKAP5	rs72989577		C/T							p.Asp1675Asn	c.5023G>A
chr2:133540605	G	NCKAP5	rs13016342	G/T	G/T		G/T					p.Pro1260Gln	c.3779C>A
chr2:133541107	T	NCKAP5	rs16841277			T/A			A/A	A/A	T/A	p.Asn1093Tyr	c.3277A>T
chr2:133542105	C	NCKAP5	rs142329411							C/G		p.Ser760Thr	c.2279G>C
chr2:133542585	C	NCKAP5	rs17325719	C/G	C/G		C/G					p.Ser600Thr	c.1799G>C
chr2:206480353	C	PARD3B	rs80119103						C/A			p.Pro1083His	c.3248C>A
chr5:7520881	G	ADCY2	rs13166360			G/T					G/T	p.Val147Leu	c.439G>T
chr5:52201722	C	ITGA1	rs4145748				C/T					p.Thr480Met	c.1439C>T
chr5:52229745	T	ITGA1	rs12520591				T/G					p.Ile961Met	c.2883T>G
chr5:52243247	G	ITGA1	Novel							G/T		p.Ala1151Ser	c.3451G>T
chr5:78076462	G	ARSB	Novel		G/A							p.Pro454Ser	c.1360C>T
chr5:132561468	C	FSTL4	rs61741674								C/A	p.Glu353Asp	c.1059G>T
chr6:12120588	C	HIVEP1	rs2228209		C/T		C/T		C/T	C/T	C/T	p.Thr187Met	c.560C>T
chr6:32917412	G	HLA-DMA	rs17214044			G/A						p.Arg210Cys	c.628C>T
chr6:152665271	G	SYNE1	rs150179494					A/A				p.Pro4057Leu	c.12170C>T
chr7:1484457	G	MICALL2	rs61736381							G/A		p.Arg417Trp	c.1249C>T
chr8:2909992	G	CSMD1	rs6558702		G/A	A/A	G/A	G/A		G/A		p.Thr2551Met	c.7652C>T
chr8:72977703	C	TRPA1	rs920829	C/T		C/T	C/T	C/T	C/T			p.Glu179Lys	c.535G>A
chr9:1056870	C	DMRT2	rs41311430			C/T						p.Ala428Val	c.1283C>T
chr9:1056959	G	DMRT2	rs17641078	G/C	G/C	G/C						p.Glu458Gln	c.1372G>C
chr9:12694177	G	TYRP1	rs866994632				G/A					p.Gly61Arg	c.181G>A
chr11:63974966	G	FERMT3	rs149000560	G/A			G/A		G/A	G/A		p.Gly44Arg	c.130G>A
chr12:89745477	C	DUSP6	rs2279574		C/A	C/A				C/A	C/A	p.Val114Leu	c.340G>T
chr12:89916811	C	GALNT4, POC1B, POC1B-GALNT4	rs2230283		C/T	T/T	C/T		C/T	C/T	C/T	p.?, p.Val506Ile, p.Val334Ile	c.100+2086G>A, c.1516G>A, c.1000G>A
chr12:100042040	C	FAM71C, ANKS1B	rs11109968			C/G		G/G		G/G		p.?, p.Arg30Gly	c.1272+6805G>C, c.88C>G
chr12:106460938	G	NUAK1	rs3741883						G/C	G/C	G/C	p.Pro543Arg	c.1628C>G
chr13:24798055	G	SPATA13	Novel					G/A				p.Ala330Thr	c.988G>A
chr13:24823699	G	SPATA13	rs41287016								G/A	p.Gly580Ser	c.1738G>A
chr13:29898768	A	MTUS2	rs928661	C/C	C/C	C/C	C/C	C/C	C/C	A/C	C/C	p.Gln952Pro	c.2855A>C
chr15:68628163	C	ITGA11	rs2306022						C/T			p.Val433Met	c.1297G>A
chr15:81598269	T	IL16	rs11556218				T/G					p.Asn1147Lys	c.3441T>G
chr16:23634293	C	PALB2	rs45551636						C/T			p.Gly998Glu	c.2993G>A
chr16:81211496	C	PKD1L2	rs9935113	C/A				C/A			C/A	p.Gly785Cys	c.2353G>T
chr16:81232336	T	PKD1L2	rs34276551					T/C				p.Thr492Ala	c.1474A>G
chr16:81241100	G	PKD1L2	rs11150370, rs386792899		G/C			G/C	G/C	G/C	G/C	p.Pro301Ala	c.901C>G
chr16:81249927	C	PKD1L2	rs7185774	C/T	C/T	T/T	T/T			T/T		p.Gly129Asp	c.386G>A
chr16:81249954	T	PKD1L2	rs7191351	A/A	A/A	A/A	A/A	T/A		A/A		p.Gln120Leu	c.359A>T
chr16:82660738	C	CDH13	rs753448816						C/A			p.Pro26Thr	c.76C>A
chr17:3632836	G	ITGAE	rs1716		G/A	G/A		G/A		G/A	G/A	p.Arg950Trp	c.2848C>T
chr17:3657159	C	ITGAE	rs2272606	C/T	C/T	C/T	C/T	T/T	T/T		C/T	p.Arg482Gln	c.1445G>A
chr17:3661052	G	ITGAE	rs71366574				G/A					p.Thr323Met	c.968C>T
chr19:52497745	T	ZNF615	rs77230420	T/C	T/C							p.Gln206Arg	c.617A>G

Table 3 and Supplementary Table S2 show genes related to autism in all patients (1–8). Six variants (chr1:98165091, chr6:32029183, chr6:32035603, chr6:32064098, chr8:2909992, chr16:84213434) were found in three out of four patients with both ADHD and autism, indicating that these genes may have a possible role in causing autism, with the exception of the chr16:84213434 variant, which was also found in three non-autistic ADHD patients, and the chr8:2909992 variant, which was also found in two non-autistic ADHD patients, which shows a stronger link with ADHD and indicates that autism in this case was a complication of ADHD. Eight variants (chr6:31602967, chr6:32010272, chr6:32063681, chr6:32064372, chr6:32065023, chr6:43323852, chr16:84213684, chr19:52497745) were found in two patients with ADHD and autism; however, the chr6:32064372 variant was found in one non-autistic ADHD patient, and the chr6:32010272 variant was also found in all four non-autistic ADHD patients, showing a more frequent association with ADHD than with autism.

**TABLE 3:** Rare and novel variants of autism genes identified by exome sequencing in all 8 Saudi Arabian ADHD non-autistic and ADHD autistic patients.

**Locus**	**Ref**	**Genes**	**rs ID number**	**Samples**								**Amino Acid Change**	**Coding**
				**1**	**2**	**3**	**4**	**5**	**6**	**7**	**8**		
				**Combined ADHD and-Autism**				**ADHD Non Autistic**					
chr1:98165091	T	DPYD	rs2297595	T/C		T/C	T/C			C/C		p.Met166Val	c.496A>G
chr1:115222237	T	AMPD1	rs34526199			T/A						p.Lys320Ile	c.959A>T
chr1:200968575	T	KIF21B	rs753390486								T/G	p.Glu596Ala	c.1787A>C
chr2:206480353	C	PARD3B	rs80119103						C/A			p.Pro1083His	c.3248C>A
chr2:233745908	C	NGEF	Novel			C/G		C/G				p.Glu630Asp	c.1890G>C
chr4:102751014	G	BANK1	rs35978636	G/C									
chr4:170618449	T	CLCN3	Novel	T/C				T/C		T/C		p.Leu376Pro	c.1127T>C
chr5:45645349	G	HCN1	Novel				G/C					p.Leu263Val	c.787C>G
chr6:31602967	G	PRRC2A	rs1046089	G/A			G/A					p.Arg1740His	c.5219G>A
chr6:31604591	C	PRRC2A	rs10885	C/T									
chr6:31852461	AG	EHMT2	Novel	A/GA						A/GA		p.Leu855Ser, p.Leu855fs	c.2563_ 2564delCTinsTC, c.2563delC
chr6:32010272	T	TNXB, CYP21A2	rs17421133	T/A	T/A			T/A	T/A	T/A	T/A	p.?, p.Asn4055Ile	c.*1361T>A, c.12164A>T
chr6:32012987	A	TNXB	rs62402693	A/GA									
chr6:32020717	G	TNXB	rs149492184								G/T	p.Pro2947Thr	c.8839C>A
chr6:32023903	G	TNXB	rs440160	G/C					G/C			p.Pro2731Arg	c.8192C>G
chr6:32029183	C	TNXB	rs2269429		C/T	C/T	C/T			C/T		p.Gly2495Ser	c.7483G>A
chr6:32035603	C	TNXB	rs9469081		C/T	C/T	T/T			C/T		p.Val2127Met	c.6379G>A
chr6:32063681	C	TNXB	rs17201602, rs751465994		C/T		C/T					p.Arg650His	c.1949G>A
chr6:32064098	C	TNXB	rs204896		C/T	C/T	T/T			C/T		p.Arg511His	c.1532G>A
chr6:32064372	C	TNXB	rs780536658		C/T	C/T					C/T	p.Val420Met	c.1258G>A
chr6:32065023	C	TNXB	rs41270461		C/T		C/T					p.Val203Met	c.607G>A
chr6:32166736	C	NOTCH4	rs867581638					C/T				p.Arg1501Gln	c.4502G>A
chr6:32363893	G	BTNL2	rs28362679		G/A							p.Ser334Leu	c.1001C>T
chr6:32364052	C	BTNL2	rs41355746								C/T	p.Arg281Lys	c.842G>A
chr6:32917412	G	HLA-DMA	rs17214044			G/A						p.Arg210Cys	c.628C>T
chr6:43323717	T	ZNF318	Novel								T/C	p.Gln452Arg	c.1355A>G
chr6:43323852	C	ZNF318	rs34541323	C/A	C/A							p.Ser407Ile	c.1220G>T
chr6:72596742	G	RIMS1	Novel							G/C		p.Gly6Arg	c.16G>C
chr6:84333028	G	SNAP91	rs749414698		G/A							p.Leu267Phe	c.799C>T
chr6:152665271	G	SYNE1	rs150179494					A/A				p.Pro4057Leu	c.12170C>T
chr7:70255653	G	AUTS2	Novel						G/A			p.Glu1151Lys	c.3451G>A
chr8:2909992	G	CSMD1	rs6558702		G/A	A/A	G/A	G/A		G/A		p.Thr2551Met	c.7652C>T
chr8:27361241	C	EPHX2	rs17057255	C/T				C/T		C/T		p.Arg103Cys	c.307C>T
chr8:27373865	G	EPHX2	rs751141							G/A		p.Arg287Gln	c.860G>A
chr8:143399990	G	TSNARE1	rs374364548	G/A								p.Thr300Met	c.899C>T
chr11:130750592	G	SNX19	rs142783173		G/A							p.Arg895Trp	c.2683C>T
chr11:130784396	G	SNX19	rs62621284							A/A	G/A	p.Pro480Leu	c.1439C>T
chr11:130784574	C	SNX19	rs62642497			T/T						p.Gly421Arg	c.1261G>A
chr12:57618909	CA	NXPH4	Novel							C/GC		p.Lys103Gln, p.Lys104fs	c.306_ 307delCAinsGC, c.311delA
chr12:100042040	C	FAM71C, ANKS1B	rs11109968			C/G		G/G		G/G		p.?, p.Arg30Gly	c.1272+6805G>C, c.88C>G
chr15:85400566	C	ALPK3	rs35292668	C/T					T/T			p.Thr1068Met	c.3203C>T
chr15:85405995	T	ALPK3	rs187316						C/C		T/C	p.Leu1622Pro	c.4865T>C
chr15:91419548	C	FURIN	rs148110342								C/T	p.Arg81Cys	c.241C>T
chr16:23634293	C	PALB2	rs45551636						C/T			p.Gly998Glu	c.2993G>A
chr16:84213434	A	TAF1C	rs2230129		T/T	A/T	T/T	T/T	A/T	A/T		p.Leu549Met	c.1645T>A
chr16:84213684	C	TAF1C	rs4150167		C/T		C/T					p.Gly497Arg	c.1489G>A
chr16:88779132	C	CTU2	rs2290895								C/T	p.His186Tyr	c.556C>T
chr16:88951637	C	CBFA2T3, LOC101927793	rs185715659					C/T				p.Glu312Lys	c.934G>A
chr18:5419723	C	EPB41L3	rs117538203			C/T						p.Arg498Gln	c.1493G>A
chr19:38901633	C	RASGRP4	rs34377632					C/T				p.Glu620Lys	c.1858G>A
chr19:52497745	T	ZNF615	rs77230420	T/C	T/C							p.Gln206Arg	c.617A>G
chr22:40075733	C	CACNA1I	rs56656729		C/A							p.Leu1766Met	c.5296C>A

Table 4 and Supplementary Table S3 show genes that play a role in both autism and ADHD. As shown in this table, the chr8:2909992 variant was found in five out of eight patients, and three of those patients had both ADHD and autism. This variant might be related to increased complications and severity of ADHD. The chr12:100042040 variant was found in three of the eight patients, one of them with both ADHD and autism. This variant could be related to the severity of ADHD, but this needs more investigation. The chr2:116525960, chr15:68624396, chr15:91452595, chr15:92647645, and chr16:82673047 variants were found in all the patients of both groups (ADHD and ADHD with autism); these might be in common variants found in ADHD patients. Likewise, the chr5:26988328 and chr15:68605169 variants were found in 7/8 patients, the chr5:26988424 variant was found in 6/8 patients, the chr7:73470714 variant was found in 5/8 patients, and the chr2:116510817 and chr9:1056728 variants were found in 4/8 patients. All of these variants may be common in ADHD and therefore do not have a link with autism or the severity of ADHD. However, the chr9:1056959 variant was found in only three of the patients with ADHD and autism; thus, this variant might be very important for the severity of ADHD and may have an association with autism.

**TABLE 4:** The identified SNPs from all study participants that were related to both autism and ADHD.

**Locus**	**Ref**	**Genes**	**rs ID number**	**1**	**2**	**3**	**4**	**5**	**6**	**7**	**8**	**Coding**	**Amino Acid Change**
		
				**Combined ADHD and-Autism**				**ADHD Non Autistic**					
chr1:200968575	T	KIF21B	rs753390486								T/G	c.1787A>C	p.Glu596Ala
chr2:116503671	G	DPP10	rs36044503				G/A	G/A				c.874G>A	p.Val292Met
chr2:116510817	G	DPP10	rs2053724	G/C		C/C		G/C			G/C	c.1030G>C	p.Ala344Pro
chr2:116525960	G	DPP10	rs1446495	A/A	A/A	A/A	A/A	A/A	A/A	A/A	A/A	c.1213G>A	p.Val405Ile
chr2:206480353	C	PARD3B	rs80119103						C/A			c.3248C>A	p.Pro1083His
chr5:26988328	G	CDH9	rs2288466	G/A		A/A	G/A	A/A	G/A	A/A	G/A	c.113C>T	p.Ala38Val
chr5:26988424	T	CDH9	rs2288467			C/C	T/C	C/C	T/C	C/C	T/C	c.17A>G	p.Tyr6Cys
chr6:32917412	G	HLA-DMA	rs17214044			G/A						c.628C>T	p.Arg210Cys
chr6:152665271	G	SYNE1	rs150179494					A/A				c.12170C>T	p.Pro4057Leu
chr7:73470714	G	ELN	rs2071307	G/A	G/A		G/A	G/A	G/A			c.1264G>A	p.Gly422Ser
chr8:2909992	G	CSMD1	rs6558702		G/A	A/A	G/A	G/A		G/A		c.7652C>T	p.Thr2551Met
chr9:1056728	G	DMRT2	rs3824419				C/C	C/C	C/C	G/C		c.1141G>C	p.Ala381Pro
chr9:1056870	C	DMRT2	rs41311430			C/T						c.1283C>T	p.Ala428Val
chr9:1056959	G	DMRT2	rs17641078	G/C	G/C	G/C						c.1372G>C	p.Glu458Gln
chr12:100042040	C	FAM71C, ANKS1B	rs11109968			C/G		G/G		G/G		c.1272+6805G>C, c.88C>G	p.?, p.Arg30Gly
chr13:95034749	G	GPC6	rs1535692					G/A		G/A	G/A	c.1234G>A	p.Val412Met
chr15:68605169	G	ITGA11	rs4777035	G/A		G/A	G/A	G/A	G/A	A/A	G/A	c.2915C>T	p.Pro972Leu
chr15:68624396	A	ITGA11	rs7168069	C/C	C/C	C/C	C/C	C/C	A/C	A/C	C/C	c.1571T>G	p.Leu524Arg
chr15:68628049	T	ITGA11	rs2306024	T/G								c.1411A>C	p.Met471Leu
chr15:68628163	C	ITGA11	rs2306022						C/T			c.1297G>A	p.Val433Met
chr15:91448626	C	MAN2A2	rs28446956				C/T		C/T			c.278C>T	p.Thr93Met
chr15:91452595	A	MAN2A2	rs2106673	A/G	G/G	A/G	A/G	A/G	A/G	A/G	A/G	c.1235A>G	p.Gln412Arg
chr15:91459475	G	MAN2A2	rs12909056				G/A					c.2983G>A	p.Val995Met
chr15:92647645	G	SLCO3A1	rs1517618	C/C	C/C	C/C	C/C	C/C	C/C	C/C	C/C	c.882G>C	p.Glu294Asp
chr16:23634293	C	PALB2	rs45551636						C/T			c.2993G>A	p.Gly998Glu
chr16:82660738	C	CDH13	rs753448816						C/A			c.76C>A	p.Pro26Thr
chr16:82673047	C	CDH13	rs4782724	T/T	T/T	T/T	T/T	T/T	T/T	T/T	T/T	c.163C>T	p.Pro55Ser
chr16:83065791	G	CDH13	rs200199969	G/A								c.475G>A	p.Val159Ile
chr17:28545280	T	SLC6A4	rs753300083								T/C	c.554A>G	p.Tyr185Cys
chr19:52497745	T	ZNF615	rs77230420	T/C	T/C							c.617A>G	p.Gln206Arg

## Discussion

The results from this study showed SNPs in several genes with variable frequencies. SNPs in the CSMD1, DUSP6 and PKD1L2 genes showed higher frequencies among all identified gene SNPs. Variations in the latter gene (polycystic kidney disease 1-like 2) showed a strong association with aggressiveness in ADHD patients. This finding was in concordance with the results of a study by Brevik et al. (2016). CSMD1 was stated as one of the candidate genes responsible for causing ADHD (Franke et al., 2009), and the results or our study support this statement. A previous exome sequencing study revealed that alterations in CSMD1 were associated with autism, which indicates the involvement of this gene in other psychological disorders (Cukier et al., 2014). Another gene that showed a correlation with neurological deficits is DUSP6. This gene is responsible for the synthesis of one of the Class I classical cysteine-based protein phosphatases. Variations in DUSP6 may have an influence on neuronal proteostasis and could lead to glutamate-induced cytotoxicity (Bhore et al., 2017). Another study detected an association between DUSP6 and bipolar disorder and highlighted the impact of DUSP6 on the therapeutic effects of lithium (Kim et al., 2012). In addition, DUSP6 could influence dopamine levels in neural synapses, which have an effect on the regulation of neurotransmitter homeostasis. Because ADHD is affected by dopamine levels, alterations in ADHD symptoms could be associated with DUSP6 variations (Demontis et al., 2017). Among all the genes with high frequencies of variation in ADHD patients, MTUS2 showed the highest frequency. There are few studies that suggest the association of MTUS with psychological disorders. MTUS, also known as KIAA0774, was found to be expressed in both the heart and the nervous system. Thus, it potentially has an effect on neurological functions (Du Puy et al., 2009). Alterations in this gene could contribute to the development of ADHD, and this finding was indicated in the results from this study. On the other hand, there are few genes that had less frequency of variation in ADHD patients, including RERE, ITGA1 and CDH13. A study indicated that mutations in the RERE gene can lead to an autosomal-dominant genetic syndrome. Symptoms of this syndrome include seizures, developmental defects in neurons, hypotonia and structural CNS abnormalities (Fregeau et al., 2016). The findings from the previous study suggested that alterations in RERE could affect neurological functions, which may lead to ADHD, autism and other neuro-developmental disorders. The results from this study showed an association between RERE variations and ADHD. In a study that was done on Han Chinese ADHD patients, the researchers detected SNPs in the ITGA gene and associated these SNPs with ADHD (Liu et al., 2017). This conclusion was confirmed by the results of this study. Finally, the CDH13 gene has an important role in the regulation of neural cell growth. A linear regression analysis in a previous study indicated that an SNP in CDH13 has a strong association with verbal working memory performance, which has a proven correlation with ADHD (Arias-Vasquez et al., 2011). In addition, deletions in CDH13 were associated with autism, suggesting a role of CHD13 in the development of psychological disorders (Sanders et al., 2015). The findings from this study presented an association between CDH13 variations and ADHD.

Autism spectrum disorder is a neurological disorder with high heritability, and approximately 18 in 10,000 children are affected in Saudi Arabia (Alnemary et al., 2017; Johannessen et al., 2017). Currently, the main causative genetic mutations of ASD still seem to be unrecognized, and more advanced studies are strongly needed to fully understand the causes of ASD. Because ASD and ADHD both have high heritability, their development might share the same genetic roots (Miles, 2011). Indeed, the investigation of this assumption could shed light on several aspects of the mechanism of the development of ASD and ADHD, with the objective to determine their common causative agent.

Here, in our study, we tested genetic variants in eight children with ADHD. Four of these children had ASD associated with ADHD. To identify the unique SNPs in the four children with ADHD and ASD, we compared the SNPs found in these children with those of the four children with only ADHD as a background control. Our data show that both chr6:43323852 and chr19:52497745 SNPs were exclusively found in the children with ADHD and ASD, identified in only 50% of these patients. These SNPs were found in the ZNF318 and ZNF615 genes, respectively. Researchers have reported the association of these gene mutations with some psychiatric disorders (e.g., ASD and bipolar disorder) (Priebe et al., 2011). Here, using the same comparison criteria, we noticed common mutations in our study groups.

Our comparison analysis shows that SNPs in the CSMD1, EPHX2, FAM71C, ANKS1B, TAF1C, DPYD, TNXB, CYP21A2, and TNXB genes overlapped. This finding might give insight into genetic mutations that cause both ASD and ADHD and support the previous hypothesis of sharing similar genetic roots (Miles, 2011). Another possible interpretation is that these SNPs might not be directly related to these medical conditions and might exist in the Saudi Arabian general population. Despite this possibility, numerous studies have found a strong association between mutations in these genes and psychiatric diseases (Liu et al., 2004; Brecevic et al., 2015; Athanasiu et al., 2017).

In agreement with the literature data, our sequencing analysis indicated an association between autosomal homozygosity and ASD/ADHD. We observed that SNPs in the DPYD, CSMD1, TAF1C and FAM71C genes might be affected by homozygosity. It is well known that homozygosity increases the susceptibility to ASD (Gamsiz et al., 2013). Our study argues that homozygosity/heterogeneity might contribute to ASD, either preventing it or conferring susceptibility to the disease. However, few studies have addressed this hypothesis, and more elaboration and studies with larger sample size are highly recommended.

As Table 4 shows, patients were divided into two groups, patients 1–4 suffered from ADHD and autism (Group 1), whereas patients 5–8 had only ADHD (Group 2). SNPs that appeared only in patients with only ADHD (Group 2) are chr1:200968575, located on the KIF21B gene; chr2:206480353, located on the PARD3B gene; chr6:152665271, located on the SYNE1 gene; and chr16:23634293, located on the PALB2 gene, with a low variant frequency (0.12) compared to that of other SNPs detected in this study. The SYNE1 gene was identified among the top ten risk loci in the most recent and largest genome-wide association study for the identification of genes conferring risk for the five major psychiatric disorders: major depression, schizophrenia, bipolar disorder (BD), autism and ADHD (Cross-Disorder Group of the Psychiatric Genomics Consortium, 2013). However, this study suggests that the SYNE1 gene is more frequently associated with ADHD than with autism. Previous studies have also shown evidence that SYNE1 is affected in bipolar disorder (Xu et al., 2014). Studies have found that the adult ADHD phenotype is commonly reported by individuals with BD (McIntyre et al., 2010) and that children with ADHD have a higher risk of being diagnosed with BD later in life (Wang et al., 2016). Our result suggests that the KIF21B gene may have a role in the development of ADHD. Genetic variation in some members of the kinesin family, to which KIF21B the gene belongs, has been associated with neurodegenerative diseases such as Alzheimer’s disease, amyotrophic lateral sclerosis (ALS) and Huntington’s disease (Hirokawa et al., 2010). The KIF21B gene, which is highly expressed in the CNS, mostly in the dendrites of neurons (Marszalek et al., 1999), was previously reported to be associated with multiple sclerosis (MS) (Wang et al., 2010). The chr16:23634293 SNP in the PALB2 gene was involved in increasing the risk of familial breast cancer in a previous study (Thompson et al., 2015). The PALB2 gene has been associated with Fanconi anemia (Sluiter et al., 2009), pancreatic cancer and hereditary breast cancer (Rahman et al., 2007; Hofstatter et al., 2011; Blanco et al., 2013). The chr12:100042040 SNP, located on the FAM71C, ANK51B gene, was found in both Group 1 and Group 2, as shown in Table 4, with a variant frequency of 0.38, demonstrating the possibility that patients may have other complications with ADHD.

This study identified the chr6:32917412 SNP, located on the HLA-DMA gene, in patient 3 of group 1. Human leukocyte antigens (HLAs) are the major histocompatibility complex (MHC) proteins in humans. The MHC complex consists of more than 200 genes located on chromosome 6. These genes are categorized into 3 classes based on their functions: class I, class II, and class III. They are involved in many biological processes, such as acting as ligands for immune cell receptors, inflammation and histocompatibility (Torres et al., 2012). Furthermore, HLA genes plays a role in pregnancy maintenance and reproduction (Knapp, 2005; Ziegler et al., 2005) and have been associated with many diseases/disorders, including autism. The importance of the HLA genes in autism was suggested by Stubbs and Magenis (1980). The association of the HLA region with autism was first reported by Warren et al. (1991), who indicated that the HLA ancestral haplotype 44.1 (B44-SC30-DR4) was associated with autism, with a relative risk of 7.9. This connection between the HLA genes and autism has been observed in all HLA classes. For example, two studies, done by Ferrante et al. (2003) and Torres et al. (2006), revealed the association of the HLAA2 gene (which belongs to the class I region) with autism, and it has been reported that the DRβ allele in the class II region is associated with autism (Torres et al., 2002; Lee et al., 2006; Johnson et al., 2009). In addition, several studies have noted an association of autism with the C4B null allele in the class III region, with relative risks of 4.6 (Warren et al., 1996) and 4.3 (Odell et al., 2005).

These previous studies support our findings suggesting that the HLA-DMA gene in the class II region might be associated with autism. In the same degree, our results suggest that the rs77230420 SNP, located on the ZNF615 gene, has a possible role in autism because it appears only in patients 1 and 2 of group 1 (patients with ADHD and autism), as Table 4 indicates.

The chr8:2909992 SNP, located in the third intron of the CUB and Sushi multiple domains 1 (CSMD1) gene, appeared in 5 of the 8 total patients; this SNP had a high variant frequency of 0.62, as demonstrated in Table 4, and a SIFT of 0.04, predicting a damaging functional effect. The CSMD1 gene is a complement control-related gene that is composed of 48 exons and covers 2 Mb of DNA. It is expressed throughout the body and has been related to many diseases, such as Kawasaki disease (Burgner et al., 2009), asthma (Hardin et al., 2014), and colon cancer (Farrell et al., 2008). CSMD1 function in the brain is not clear. However, as part of the brain complement cascade, CSMD1 is involved in the regulation of synaptic function (Kraus et al., 2006). A wide range of previous studies have confirmed an association of CSMD1 with schizophrenia (Havik et al., 2011; Kwon et al., 2013). CSMD1 has also been reported to be associated with bipolar disorder (Xu et al., 2014) and cannabis dependence (Sherva et al., 2016). Furthermore, rare variants in the CSMD1 gene have been reported to be involved in autism (Cukier et al., 2014). Finally, a study done by Athanasiu et al. (2017) on 3437 individuals found evidence that a variant in the CSMD1 gene is associated with cognitive function. These findings, in addition to our results, suggest that many variants in CSMD1 may be involved in various phenotypes related to brain dysfunction.

### Strengths and Limitations

The strength of our current study is that we selected patients with an accurate diagnosis of the severity of ADHD from the same region with multiple assessment protocols.

Our study was conducted in a relatively small subpopulation in the western region of Saudi Arabia, and the generalizability of the current results to other Saudi populations may require a further complementary study with a larger sample size from other regions of Saudi Arabia. Some limitations were detected in the current study; (i) There was a limited number of patients. Further investigations including age-matched controls and a large number of patients are necessary to confirm the associations of the various SNPs in Saudi children with ADHD. (ii) Some complications associated with ADHD, such as learning disorders (dyslexia), were not controlled in this study.

### Recommendations

Our study results recommend the performance of screening tests for the SNPs identified in ASD associated with ADHD in pediatric patients to confirm their prevalence in the Saudi population. It is also recommended for future studies to examine the 11 SNP variants in detail.

## Data Availability Statement

The datasets for this article are not publicly available due to concerns regarding participant/patient anonymity. Requests to access the datasets should be directed to the corresponding author.

## Ethics Statement

The studies involving human participants were reviewed and approved by the Medical Ethical Committee of the Faculty of Medicine, Umm Al-Qura University (Ethical approval: written informed consent to participate in this study was provided by the participants’ legal guardian/next of kin). Written informed consent was obtained from the legal guardians of the participants for the publication of any potentially identifiable data included in this article.

## Author Contributions

NMB, AAA, MMT, and FAA conceived and planned the study. NAQ, SA, AmA, and DMA contributed to patient recruitment and clinical data collection. SA, AsA, GD, and HSB performed the experiments. AAA and NMB performed the statistical analyses. NMB, AAA, and MMT drafted the manuscript and evaluated for important intellectual content. FAA edited and revised the manuscript. NMB, AAA, and MMT supervised the study. All authors critically reviewed the manuscript and approved the final version of the manuscript to be published.

## Conflict of Interest

AmA was employed by the company N.Sight Brain Training and Consultation Center which is classified as the first company of its kind in the Kingdom of Saudi Arabia that specializes in developing cognitive skills based on customized one-on-one brain training program. The remaining authors declare that the research was conducted in the absence of any commercial or financial relationships that could be construed as a potential conflict of interest.

## Supplementary Material

Supplementary Table 1 Rare and novel variants of ADHD genes identified by exome sequencing in the 8 Saudi Arabian ADHD patients.

Supplementary Table 2 Rare and novel variants of autism genes identified by exome sequencing in all 8 Saudi Arabian ADHD non-autistic and ADHD autistic patients.

Supplementary Table 3 The identified SNPs from all study participants that were related to both autism and ADHD.

FIGURE S1 Lists of the genes used in filtration.
