# Supplementary material for: The Co-existence of ADHD With Autism in Saudi Children: An Analysis Using Next-Generation DNA Sequencing
Source: Front Genet. 2020 Dec 15;11:548559. doi: 10.3389/fgene.2020.548559 (PMC7770135; doi:10.3389/fgene.2020.548559)
Supplement: Supplementary Table 1 — Rare and novel variants of ADHD genes identified by exome sequencing in the 8 Saudi Arabian ADHD patients. [file Data_Sheet_1.docx]

**Supplements**

**Supplements**

Supplement 1. Rare and novel variants of ADHD genes identified by exome sequencing in the 8 Saudi Arabian ADHD patients

| Locus | Ref | dbSNP | Variant Frequency | Genes | 1 | 2 | 3 | 4 | 5 | 6 | 7 | 8 | Amino Acid Change | Coding | Exon | PhyloP | SIFT | Grantham | PolyPhen |
| --- | --- | --- | --- | --- | --- | --- | --- | --- | --- | --- | --- | --- | --- | --- | --- | --- | --- | --- | --- |
| chr1:70505307 | A | Novel | 0.12 | LRRC7 |  |  | A/G |  |  |  |  |  | p.Glu1229Gly | c.3686A>G | 19 | 8.82 | 0 | 98 | 0.888 |
| chr1:70541953 | A | Novel | 0.12 | LRRC7 |  |  | A/C |  |  |  |  |  | p.Gln1437Pro | c.4310A>C | 22 | 7.37 | 0 | 76 | 0.99 |
| chr1:200968575 | T | rs753390486 | 0.12 | KIF21B |  |  |  |  |  |  |  | T/G | p.Glu596Ala | c.1787A>C | 13 | 6.97 | 0.03 | 107 | 0.993 |
| chr1:218520315 | G | rs10482721 | 0.12 | TGFB2 |  | G/A |  |  |  |  |  |  | p.Arg91His | c.272G>A | 1 | 6.55 | 0.02 | 29 | 0.995 |
| chr2:111598958 | C | rs1554005 | 0.12 | ACOXL |  |  |  |  |  |  |  | C/T | p.Thr255Met | c.764C>T | 10 | 0.15 | 0 | 81 | 0.672 |
| chr2:111850515 | C | rs17041850 | 0.12 | ACOXL |  |  |  |  |  | C/T |  |  | p.Pro505Leu | c.1514C>T | 17 | 2.04 | 0.03 | 98 | 0.978 |
| chr2:133489376 | C | rs776162189 | 0.12 | NCKAP5 |  |  |  |  |  | C/G |  |  | p.Asp1793His | c.5377G>C | 17 | 6.14 | 0 | 81 | 1 |
| chr2:133538651 | C | rs72989577 | 0.12 | NCKAP5 |  | C/T |  |  |  |  |  |  | p.Asp1675Asn | c.5023G>A | 15 | 2.32 | 0.01 | 23 | 0.76 |
| chr2:133540605 | G | rs13016342 | 0.38 | NCKAP5 | G/T | G/T |  | G/T |  |  |  |  | p.Pro1260Gln | c.3779C>A | 14 | 6.5 | 0 | 76 | 0.996 |
| chr2:133541107 | T | rs16841277 | 0.5 | NCKAP5 |  |  | T/A |  |  | A/A | A/A | T/A | p.Asn1093Tyr | c.3277A>T | 14 | 0.5 | 0 | 143 | 0.969 |
| chr2:133542105 | C | rs142329411 | 0.12 | NCKAP5 |  |  |  |  |  |  | C/G |  | p.Ser760Thr | c.2279G>C | 14 | -0.69 | 0.02 | 58 | 0.589 |
| chr2:133542585 | C | rs17325719 | 0.38 | NCKAP5 | C/G | C/G |  | C/G |  |  |  |  | p.Ser600Thr | c.1799G>C | 14 | 5.76 | 0 | 58 | 0.882 |
| chr2:206480353 | C | rs80119103 | 0.12 | PARD3B |  |  |  |  |  | C/A |  |  | p.Pro1083His | c.3248C>A | 22 | 1.09 | 0 | 77 | 0.606 |
| chr5:7520881 | G | rs13166360 | 0.25 | ADCY2 |  |  | G/T |  |  |  |  | G/T | p.Val147Leu | c.439G>T | 3 | 6 | 0 | 32 | 0.598 |
| chr5:52201722 | C | rs4145748 | 0.12 | ITGA1 |  |  |  | C/T |  |  |  |  | p.Thr480Met | c.1439C>T | 12 | 1.79 | 0.01 | 81 | 0.941 |
| chr5:52229745 | T | rs12520591 | 0.12 | ITGA1 |  |  |  | T/G |  |  |  |  | p.Ile961Met | c.2883T>G | 23 | 2.32 | 0.04 | 10 | 0.91 |
| chr5:52243247 | G | Novel | 0.12 | ITGA1 |  |  |  |  |  |  | G/T |  | p.Ala1151Ser | c.3451G>T | 28 | 3.52 | 0.03 | 99 | 0.998 |
| chr5:78076462 | G | Novel | 0.12 | ARSB |  | G/A |  |  |  |  |  |  | p.Pro454Ser | c.1360C>T | 8 | 7.94 | 0.02 | 74 | 0.847 |
| chr5:132561468 | C | rs61741674 | 0.12 | FSTL4 |  |  |  |  |  |  |  | C/A | p.Glu353Asp | c.1059G>T | 9 | 0.58 | 0 | 45 | 0.999 |
| chr6:12120588 | C | rs2228209 | 0.62 | HIVEP1 |  | C/T |  | C/T |  | C/T | C/T | C/T | p.Thr187Met | c.560C>T | 4 | 0.64 | 0 | 81 | 0.982 |
| chr6:32917412 | G | rs17214044 | 0.12 | HLA-DMA |  |  | G/A |  |  |  |  |  | p.Arg210Cys | c.628C>T | 3 | -1.09 | 0.01 | 180 | 0.996 |
| chr6:152665271 | G | rs150179494 | 0.12 | SYNE1 |  |  |  |  | A/A |  |  |  | p.Pro4057Leu | c.12170C>T | 74 | 6.51 | 0.01 | 98 | 0.936 |
| chr7:1484457 | G | rs61736381 | 0.12 | MICALL2 |  |  |  |  |  |  | G/A |  | p.Arg417Trp | c.1249C>T | 6 | 2.79 | 0 | 101 | 1 |
| chr8:2909992 | G | rs6558702 | 0.62 | CSMD1 |  | G/A | A/A | G/A | G/A |  | G/A |  | p.Thr2551Met | c.7652C>T | 50 | 0.96 | 0.04 | 81 | 0.653 |
| chr8:72977703 | C | rs920829 | 0.62 | TRPA1 | C/T |  | C/T | C/T | C/T | C/T |  |  | p.Glu179Lys | c.535G>A | 4 | 6.72 | 0.05 | 56 | 0.994 |
| chr9:1056870 | C | rs41311430 | 0.12 | DMRT2 |  |  | C/T |  |  |  |  |  | p.Ala428Val | c.1283C>T | 4 | 7.29 | 0 | 64 | 1 |
| chr9:1056959 | G | rs17641078 | 0.38 | DMRT2 | G/C | G/C | G/C |  |  |  |  |  | p.Glu458Gln | c.1372G>C | 4 | 7.16 | 0 | 29 | 0.988 |
| chr9:12694177 | G | rs866994632 | 0.12 | TYRP1 |  |  |  | G/A |  |  |  |  | p.Gly61Arg | c.181G>A | 2 | 7.42 | 0 | 125 | 1 |
| chr11:63974966 | G | rs149000560 | 0.5 | FERMT3 | G/A |  |  | G/A |  | G/A | G/A |  | p.Gly44Arg | c.130G>A | 2 | 8.53 | 0 | 125 | 1 |
| chr12:89745477 | C | rs2279574 | 0.5 | DUSP6 |  | C/A | C/A |  |  |  | C/A | C/A | p.Val114Leu | c.340G>T | 1 | 5.81 | 0.03 | 32 | 0.78 |
| chr12:89916811 | C | rs2230283 | 0.75 | GALNT4, POC1B, POC1B-GALNT4 |  | C/T | T/T | C/T |  | C/T | C/T | C/T | p.?, p.Val506Ile, p.Val334Ile | c.100+2086G>A, c.1516G>A, c.1000G>A | 3 | 7.43 | 0.02 | 29.0, 29.0 | 0.896 |
| chr12:100042040 | C | rs11109968 | 0.38 | FAM71C, ANKS1B |  |  | C/G |  | G/G |  | G/G |  | p.?, p.Arg30Gly | c.1272+6805G>C, c.88C>G | 1 | -0.01 | 0 | 125 | 0.971 |
| chr12:106460938 | G | rs3741883 | 0.38 | NUAK1 |  |  |  |  |  | G/C | G/C | G/C | p.Pro543Arg | c.1628C>G | 7 | 3.53 | 0 | 103 | 0.93 |
| chr13:24798055 | G | Novel | 0.12 | SPATA13 |  |  |  |  | G/A |  |  |  | p.Ala330Thr | c.988G>A | 2 | 3.65 | 0.01 | 58 | 0.855 |
| chr13:24823699 | G | rs41287016 | 0.12 | SPATA13 |  |  |  |  |  |  |  | G/A | p.Gly580Ser | c.1738G>A | 3 | 1.64 | 0 | 56 | 0.817 |
| chr13:29898768 | A | rs928661 | 1 | MTUS2 | C/C | C/C | C/C | C/C | C/C | C/C | A/C | C/C | p.Gln952Pro | c.2855A>C | 5 | 1.89 | 0 | 76 | 1 |
| chr15:68628163 | C | rs2306022 | 0.12 | ITGA11 |  |  |  |  |  | C/T |  |  | p.Val433Met | c.1297G>A | 12 | -0.18 | 0.03 | 21 | 0.928 |
| chr15:81598269 | T | rs11556218 | 0.12 | IL16 |  |  |  | T/G |  |  |  |  | p.Asn1147Lys | c.3441T>G | 16 | -1.38 | 0.04 | 94 | 0.977 |
| chr16:23634293 | C | rs45551636 | 0.12 | PALB2 |  |  |  |  |  | C/T |  |  | p.Gly998Glu | c.2993G>A | 9 | 2.85 | 0 | 98 | 1 |
| chr16:81211496 | C | rs9935113 | 0.38 | PKD1L2 | C/A |  |  |  | C/A |  |  | C/A | p.Gly785Cys | c.2353G>T | 14 | 3.89 | 0 | 159 | 1 |
| chr16:81232336 | T | rs34276551 | 0.12 | PKD1L2 |  |  |  |  | T/C |  |  |  | p.Thr492Ala | c.1474A>G | 7 | 4.29 | 0.01 | 58 | 0.775 |
| chr16:81241100 | G | rs11150370,rs386792899 | 0.62 | PKD1L2 |  | G/C |  |  | G/C | G/C | G/C | G/C | p.Pro301Ala | c.901C>G | 5 | 6.05 | 0.01 | 27 | 0.951 |
| chr16:81249927 | C | rs7185774 | 0.62 | PKD1L2 | C/T | C/T | T/T | T/T |  |  | T/T |  | p.Gly129Asp | c.386G>A | 2 | 3.98 | 0.01 | 94 | 0.998 |
| chr16:81249954 | T | rs7191351 | 0.75 | PKD1L2 | A/A | A/A | A/A | A/A | T/A |  | A/A |  | p.Gln120Leu | c.359A>T | 2 | 1.17 | 0 | 113 | 0.763 |
| chr16:82660738 | C | rs753448816 | 0.12 | CDH13 |  |  |  |  |  | C/A |  |  | p.Pro26Thr | c.76C>A | 1 | 3.5 | 0 | 38 | 1 |
| chr17:3632836 | G | rs1716 | 0.62 | ITGAE |  | G/A | G/A |  | G/A |  | G/A | G/A | p.Arg950Trp | c.2848C>T | 24 | -0.21 | 0.02 | 101 | 0.999 |
| chr17:3657159 | C | rs2272606 | 0.88 | ITGAE | C/T | C/T | C/T | C/T | T/T | T/T |  | C/T | p.Arg482Gln | c.1445G>A | 13 | -0.22 | 0 | 43 | 0.783 |
| chr17:3661052 | G | rs71366574 | 0.12 | ITGAE |  |  |  | G/A |  |  |  |  | p.Thr323Met | c.968C>T | 9 | 0.35 | 0.04 | 81 | 1 |
| chr19:52497745 | T | rs77230420 | 0.25 | ZNF615 | T/C | T/C |  |  |  |  |  |  | p.Gln206Arg | c.617A>G | 7 | 0.18 | 0.02 | 43 | 0.957 |

Supplement 2. Rare and novel variants of autism genes identified by exome sequencing in all 8 Saudi Arabian ADHD non-autistic and ADHD autistic patients

| Locus | Ref | dbSNP | Variant Frequency | Genes | 1 | 2 | 3 | 4 | 5 | 6 | 7 | 8 | Amino Acid Change | Coding | Exon | PhyloP | SIFT | Grantham | PolyPhen |
| --- | --- | --- | --- | --- | --- | --- | --- | --- | --- | --- | --- | --- | --- | --- | --- | --- | --- | --- | --- |
|  |  |  |  |  | Combined ADHD and-Autism | | | | ADHD Non Autistic | | | |  |  |  |  |  |  |  |
| chr1:98165091 | T | rs2297595 | 0.5 | DPYD | T/C |  | T/C | T/C |  |  | C/C |  | p.Met166Val | c.496A>G | 6 | 7.66 | 0 | 21 | 0.999 |
| chr1:115222237 | T | rs34526199 | 0.12 | AMPD1 |  |  | T/A |  |  |  |  |  | p.Lys320Ile | c.959A>T | 7 | 8 | 0 | 102 | 1 |
| chr1:200968575 | T | rs753390486 | 0.12 | KIF21B |  |  |  |  |  |  |  | T/G | p.Glu596Ala | c.1787A>C | 13 | 6.97 | 0.03 | 107 | 0.993 |
| chr2:206480353 | C | [rs80119103](http://www.ncbi.nlm.nih.gov/projects/SNP/snp_ref.cgi?rs=rs80119103) | 0.12 | PARD3B |  |  |  |  |  | C/A |  |  | p.Pro1083His | c.3248C>A | 22 | 1.09 | 0 | 77 | 0.606 |
| chr2:233745908 | C | Novel | 0.25 | NGEF |  |  | C/G |  | C/G |  |  |  | p.Glu630Asp | c.1890G>C | 14 | 0.88 | 0 | 45 | 0.996 |
| chr4:102751014 | G | rs35978636 | 0.12 | BANK1 | G/C |  |  |  |  |  |  |  |  |  | 2 | 6.78 | 0 | 215 | 1 |
| chr4:170618449 | T | Novel | 0.38 | CLCN3 | T/C |  |  |  | T/C |  | T/C |  | p.Leu376Pro | c.1127T>C | 9 | 8.04 | 0 | 98 | 0.998 |
| chr5:45645349 | G | Novel | 0.12 | HCN1 |  |  |  | G/C |  |  |  |  | p.Leu263Val | c.787C>G | 2 | 9.87 | 0 | 32 | 0.941 |
| chr6:31602967 | G | rs1046089 | 0.25 | PRRC2A | G/A |  |  | G/A |  |  |  |  | p.Arg1740His | c.5219G>A | 22 | 3.35 | 0 | 29 | 0.957 |
| chr6:31604591 | C | rs10885 | 0.12 | PRRC2A | C/T |  |  |  |  |  |  |  |  |  | 28 | 3.08 | 0 | 74 | 0.97 |
| chr6:31852461 | AG | Novel | 0.25 | EHMT2 | A/GA |  |  |  |  |  | A/GA |  | p.Leu855Ser, p.Leu855fs | c.2563_2564delCTinsTC, c.2563delC | 20 | 9.12, 4.7 | 0 | 145 | 1 |
| chr6:32010272 | T | rs17421133 | 0.75 | TNXB, CYP21A2 | T/A | T/A |  |  | T/A | T/A | T/A | T/A | p.?, p.Asn4055Ile | c.*1361T>A, c.12164A>T | 40 | 0.05 | 0 | 149 | 0.989 |
| chr6:32012987 | A | rs62402693 | 0.12 | TNXB | A/GA |  |  |  |  |  |  |  |  |  | 32 | 1.53 | 0.01 | 74 | 0.998 |
| chr6:32020717 | G | rs149492184 | 0.12 | TNXB |  |  |  |  |  |  |  | G/T | p.Pro2947Thr | c.8839C>A | 26 | -1.47 | 0.01 | 38 | 0.8 |
| chr6:32023903 | G | rs440160 | 0.25 | TNXB | G/C |  |  |  |  | G/C |  |  | p.Pro2731Arg | c.8192C>G | 24 | 0.61 | 0.05 | 103 | 0.989 |
| chr6:32029183 | C | rs2269429 | 0.5 | TNXB |  | C/T | C/T | C/T |  |  | C/T |  | p.Gly2495Ser | c.7483G>A | 21 | 0.78 | 0.04 | 56 | 1 |
| chr6:32035603 | C | rs9469081 | 0.5 | TNXB |  | C/T | C/T | T/T |  |  | C/T |  | p.Val2127Met | c.6379G>A | 18 | -2.07 | 0.01 | 21 | 0.907 |
| chr6:32063681 | C | rs17201602,rs751465994 | 0.25 | TNXB |  | C/T |  | C/T |  |  |  |  | p.Arg650His | c.1949G>A | 3 | -1.26 | 0.01 | 29 | 1 |
| chr6:32064098 | C | rs204896 | 0.5 | TNXB |  | C/T | C/T | T/T |  |  | C/T |  | p.Arg511His | c.1532G>A | 3 | -0.04 | 0.05 | 29 | 0.585 |
| chr6:32064372 | C | rs780536658 | 0.38 | TNXB |  | C/T | C/T |  |  |  |  | C/T | p.Val420Met | c.1258G>A | 3 | 0.21 | 0 | 21 | 1 |
| chr6:32065023 | C | rs41270461 | 0.25 | TNXB |  | C/T |  | C/T |  |  |  |  | p.Val203Met | c.607G>A | 3 | 0.47 | 0 | 21 | 0.997 |
| chr6:32166736 | C | rs867581638 | 0.12 | NOTCH4 |  |  |  |  | C/T |  |  |  | p.Arg1501Gln | c.4502G>A | 24 | 5.26 | 0 | 43 | 0.663 |
| chr6:32363893 | G | rs28362679 | 0.12 | BTNL2 |  | G/A |  |  |  |  |  |  | p.Ser334Leu | c.1001C>T | 5 | 2.25 | 0.05 | 145 | 1 |
| chr6:32364052 | C | rs41355746 | 0.12 | BTNL2 |  |  |  |  |  |  |  | C/T | p.Arg281Lys | c.842G>A | 5 | 1.37 | 0.01 | 26 | 0.998 |
| chr6:32917412 | G | rs17214044 | 0.12 | HLA-DMA |  |  | G/A |  |  |  |  |  | p.Arg210Cys | c.628C>T | 3 | -1.09 | 0.01 | 180 | 0.996 |
| chr6:43323717 | T | Novel | 0.12 | ZNF318 |  |  |  |  |  |  |  | T/C | p.Gln452Arg | c.1355A>G | 4 | 5.17 | 0.01 | 43 | 0.949 |
| chr6:43323852 | C | rs34541323 | 0.25 | ZNF318 | C/A | C/A |  |  |  |  |  |  | p.Ser407Ile | c.1220G>T | 4 | 0.87 | 0 | 142 | 0.919 |
| chr6:72596742 | G | Novel | 0.12 | RIMS1 |  |  |  |  |  |  | G/C |  | p.Gly6Arg | c.16G>C | 1 | 5.98 | 0 | 125 | 1 |
| chr6:84333028 | G | rs749414698 | 0.12 | SNAP91 |  | G/A |  |  |  |  |  |  | p.Leu267Phe | c.799C>T | 9 | 5.97 | 0 | 22 | 0.999 |
| chr6:152665271 | G | rs150179494 | 0.12 | SYNE1 |  |  |  |  | A/A |  |  |  | p.Pro4057Leu | c.12170C>T | 74 | 6.51 | 0.01 | 98 | 0.936 |
| chr7:70255653 | G | Novel | 0.12 | AUTS2 |  |  |  |  |  | G/A |  |  | p.Glu1151Lys | c.3451G>A | 19 | 9.28 | 0 | 56 | 0.995 |
| chr8:2909992 | G | rs6558702 | 0.62 | CSMD1 |  | G/A | A/A | G/A | G/A |  | G/A |  | p.Thr2551Met | c.7652C>T | 50 | 0.96 | 0.04 | 81 | 0.653 |
| chr8:27361241 | C | rs17057255 | 0.38 | EPHX2 | C/T |  |  |  | C/T |  | C/T |  | p.Arg103Cys | c.307C>T | 3 | -1.59 | 0.01 | 180 | 0.985 |
| chr8:27373865 | G | rs751141 | 0.12 | EPHX2 |  |  |  |  |  |  | G/A |  | p.Arg287Gln | c.860G>A | 8 | 7.32 | 0 | 43 | 1 |
| chr8:143399990 | G | rs374364548 | 0.12 | TSNARE1 | G/A |  |  |  |  |  |  |  | p.Thr300Met | c.899C>T | 7 | 0.19 | 0.01 | 81 | 0.955 |
| chr11:130750592 | G | rs142783173 | 0.12 | SNX19 |  | G/A |  |  |  |  |  |  | p.Arg895Trp | c.2683C>T | 9 | 1.53 | 0 | 101 | 1 |
| chr11:130784396 | G | rs62621284 | 0.25 | SNX19 |  |  |  |  |  |  | A/A | G/A | p.Pro480Leu | c.1439C>T | 1 | 6.29 | 0 | 98 | 1 |
| chr11:130784574 | C | rs62642497 | 0.12 | SNX19 |  |  | T/T |  |  |  |  |  | p.Gly421Arg | c.1261G>A | 1 | 0.21 | 0 | 125 | 0.718 |
| chr12:57618909 | CA | Novel | 0.12 | NXPH4 |  |  |  |  |  |  | C/GC |  | p.Lys103Gln, p.Lys104fs | c.306_307delCAinsGC, c.311delA | 2 | 2.28, 5.67 | 0 | 53 | 1 |
| chr12:100042040 | C | rs11109968 | 0.38 | FAM71C, ANKS1B |  |  | C/G |  | G/G |  | G/G |  | p.?, p.Arg30Gly | c.1272+6805G>C, c.88C>G | 1 | -0.01 | 0 | 125 | 0.971 |
| chr15:85400566 | C | rs35292668 | 0.25 | ALPK3 | C/T |  |  |  |  | T/T |  |  | p.Thr1068Met | c.3203C>T | 6 | -0.41 | 0 | 81 | 0.865 |
| chr15:85405995 | T | rs187316 | 0.25 | ALPK3 |  |  |  |  |  | C/C |  | T/C | p.Leu1622Pro | c.4865T>C | 10 | 1.71 | 0 | 98 | 0.991 |
| chr15:91419548 | C | rs148110342 | 0.12 | FURIN |  |  |  |  |  |  |  | C/T | p.Arg81Cys | c.241C>T | 3 | 1.8 | 0.03 | 180 | 0.989 |
| chr16:23634293 | C | rs45551636 | 0.12 | PALB2 |  |  |  |  |  | C/T |  |  | p.Gly998Glu | c.2993G>A | 9 | 2.85 | 0 | 98 | 1 |
| chr16:84213434 | A | rs2230129 | 0.75 | TAF1C |  | T/T | A/T | T/T | T/T | A/T | A/T |  | p.Leu549Met | c.1645T>A | 15 | -1.93 | 0.01 | 15 | 0.899 |
| chr16:84213684 | C | rs4150167 | 0.25 | TAF1C |  | C/T |  | C/T |  |  |  |  | p.Gly497Arg | c.1489G>A | 14 | 0.35 | 0 | 125 | 0.979 |
| chr16:88779132 | C | rs2290895 | 0.12 | CTU2 |  |  |  |  |  |  |  | C/T | p.His186Tyr | c.556C>T | 7 | -0.11 | 0.02 | 83 | 0.867 |
| chr16:88951637 | C | rs185715659 | 0.12 | CBFA2T3, LOC101927793 |  |  |  |  | C/T |  |  |  | p.Glu312Lys | c.934G>A | 7 | 5.47 | 0 | 56 | 0.968 |
| chr18:5419723 | C | rs117538203 | 0.12 | EPB41L3 |  |  | C/T |  |  |  |  |  | p.Arg498Gln | c.1493G>A | 12 | 5.48 | 0 | 43 | 0.905 |
| chr19:38901633 | C | rs34377632 | 0.12 | RASGRP4 |  |  |  |  | C/T |  |  |  | p.Glu620Lys | c.1858G>A | 16 | 3.13 | 0.04 | 56 | 0.727 |
| chr19:52497745 | T | rs77230420 | 0.25 | ZNF615 | T/C | T/C |  |  |  |  |  |  | p.Gln206Arg | c.617A>G | 7 | 0.18 | 0.02 | 43 | 0.957 |
| chr22:40075733 | C | rs56656729 | 0.12 | CACNA1I |  | C/A |  |  |  |  |  |  | p.Leu1766Met | c.5296C>A | 32 | 2.15 | 0.02 | 15 | 0.998 |

Supplement 3: The identified SNPs from all study participants that were related to both autism and ADHD

| Locus | Ref | dbSNP | Variant Frequency | Genes | 1 | 2 | 3 | 4 | 5 | 6 | 7 | 8 | Coding | Exon | Amino Acid Change | PhyloP | SIFT | Grantham | PolyPhen |
| --- | --- | --- | --- | --- | --- | --- | --- | --- | --- | --- | --- | --- | --- | --- | --- | --- | --- | --- | --- |
|  |  |  |  |  | Combined ADHD and-Autism | | | | ADHD Non Autistic | | | |  |  |  |  |  |  |  |
| chr1:200968575 | T | rs753390486 | 0.12 | KIF21B |  |  |  |  |  |  |  | T/G | c.1787A>C | 13 | p.Glu596Ala | 6.97 | 0.03 | 107 | 0.993 |
| chr2:116503671 | G | rs36044503 | 0.25 | DPP10 |  |  |  | G/A | G/A |  |  |  | c.874G>A | 10 | p.Val292Met | 1.68 | 0.11 | 21 | 0.024 |
| chr2:116510817 | G | rs2053724 | 0.5 | DPP10 | G/C |  | C/C |  | G/C |  |  | G/C | c.1030G>C | 11 | p.Ala344Pro | 1.94 | 0.32 | 27 | 0 |
| chr2:116525960 | G | rs1446495 | 1 | DPP10 | A/A | A/A | A/A | A/A | A/A | A/A | A/A | A/A | c.1213G>A | 13 | p.Val405Ile | 6.28 | 1 | 29 | 0 |
| chr2:206480353 | C | rs80119103 | 0.12 | PARD3B |  |  |  |  |  | C/A |  |  | c.3248C>A | 22 | p.Pro1083His | 1.09 | 0 | 77 | 0.606 |
| chr5:26988328 | G | rs2288466 | 0.88 | CDH9 | G/A |  | A/A | G/A | A/A | G/A | A/A | G/A | c.113C>T | 2 | p.Ala38Val | 0.67 | 0.44 | 64 | 0 |
| chr5:26988424 | T | rs2288467 | 0.75 | CDH9 |  |  | C/C | T/C | C/C | T/C | C/C | T/C | c.17A>G | 2 | p.Tyr6Cys | 0.1 | 1 | 194 | 0 |
| chr6:32917412 | G | rs17214044 | 0.12 | HLA-DMA |  |  | G/A |  |  |  |  |  | c.628C>T | 3 | p.Arg210Cys | -1.09 | 0.01 | 180 | 0.996 |
| chr6:152665271 | G | rs150179494 | 0.12 | SYNE1 |  |  |  |  | A/A |  |  |  | c.12170C>T | 74 | p.Pro4057Leu | 6.51 | 0.01 | 98 | 0.936 |
| chr7:73470714 | G | rs2071307 | 0.62 | ELN | G/A | G/A |  | G/A | G/A | G/A |  |  | c.1264G>A | 20 | p.Gly422Ser | 0.53 | 0 | 56 | 0.012 |
| chr8:2909992 | G | rs6558702 | 0.62 | CSMD1 |  | G/A | A/A | G/A | G/A |  | G/A |  | c.7652C>T | 50 | p.Thr2551Met | 0.96 | 0.04 | 81 | 0.653 |
| chr9:1056728 | G | rs3824419 | 0.5 | DMRT2 |  |  |  | C/C | C/C | C/C | G/C |  | c.1141G>C | 4 | p.Ala381Pro | 0.46 | 0.25 | 27 | 0.337 |
| chr9:1056870 | C | rs41311430 | 0.12 | DMRT2 |  |  | C/T |  |  |  |  |  | c.1283C>T | 4 | p.Ala428Val | 7.29 | 0 | 64 | 1 |
| chr9:1056959 | G | rs17641078 | 0.38 | DMRT2 | G/C | G/C | G/C |  |  |  |  |  | c.1372G>C | 4 | p.Glu458Gln | 7.16 | 0 | 29 | 0.988 |
| chr12:100042040 | C | rs11109968 | 0.38 | FAM71C, ANKS1B |  |  | C/G |  | G/G |  | G/G |  | c.1272+6805G>C, c.88C>G | 1 | p.?, p.Arg30Gly | -0.01 | 0 | 125 | 0.971 |
| chr13:95034749 | G | rs1535692 | 0.38 | GPC6 |  |  |  |  | G/A |  | G/A | G/A | c.1234G>A | 7 | p.Val412Met | 5.12 | 1 | 21 | 0.112 |
| chr15:68605169 | G | rs4777035 | 0.88 | ITGA11 | G/A |  | G/A | G/A | G/A | G/A | A/A | G/A | c.2915C>T | 24 | p.Pro972Leu | 7.33 | 0 | 98 | 0.273 |
| chr15:68624396 | A | rs7168069 | 1 | ITGA11 | C/C | C/C | C/C | C/C | C/C | A/C | A/C | C/C | c.1571T>G | 14 | p.Leu524Arg | -0.13 | 0.41 | 102 | 0 |
| chr15:68628049 | T | rs2306024 | 0.12 | ITGA11 | T/G |  |  |  |  |  |  |  | c.1411A>C | 12 | p.Met471Leu | 5.49 | 1 | 15 | 0 |
| chr15:68628163 | C | rs2306022 | 0.12 | ITGA11 |  |  |  |  |  | C/T |  |  | c.1297G>A | 12 | p.Val433Met | -0.18 | 0.03 | 21 | 0.928 |
| chr15:91448626 | C | rs28446956 | 0.25 | MAN2A2 |  |  |  | C/T |  | C/T |  |  | c.278C>T | 3 | p.Thr93Met | 0.46 | 0.09 | 81 | 0.001 |
| chr15:91452595 | A | rs2106673 | 1 | MAN2A2 | A/G | G/G | A/G | A/G | A/G | A/G | A/G | A/G | c.1235A>G | 9 | p.Gln412Arg | 3.69 | 0.57 | 43 | 0 |
| chr15:91459475 | G | rs12909056 | 0.12 | MAN2A2 |  |  |  | G/A |  |  |  |  | c.2983G>A | 20 | p.Val995Met | -0.9 | 0.14 | 21 | 0.001 |
| chr15:92647645 | G | rs1517618 | 1 | SLCO3A1 | C/C | C/C | C/C | C/C | C/C | C/C | C/C | C/C | c.882G>C | 4 | p.Glu294Asp | 1.06 | 1 | 45 | 0 |
| chr16:23634293 | C | rs45551636 | 0.12 | PALB2 |  |  |  |  |  | C/T |  |  | c.2993G>A | 9 | p.Gly998Glu | 2.85 | 0 | 98 | 1 |
| chr16:82660738 | C | rs753448816 | 0.12 | CDH13 |  |  |  |  |  | C/A |  |  | c.76C>A | 1 | p.Pro26Thr | 3.5 | 0 | 38 | 1 |
| chr16:82673047 | C | rs4782724 | 1 | CDH13 | T/T | T/T | T/T | T/T | T/T | T/T | T/T | T/T | c.163C>T | 2 | p.Pro55Ser | 0.2 | 0.87 | 74 | 0 |
| chr16:83065791 | G | rs200199969 | 0.12 | CDH13 | G/A |  |  |  |  |  |  |  | c.475G>A | 4 | p.Val159Ile | 0 | 0.27 | 29 | 0.001 |
| chr17:28545280 | T | rs753300083 | 0.12 | SLC6A4 |  |  |  |  |  |  |  | T/C | c.554A>G | 5 | p.Tyr185Cys | 5.03 | 0 | 194 | 1 |
| chr19:52497745 | T | rs77230420 | 0.25 | ZNF615 | T/C | T/C |  |  |  |  |  |  | c.617A>G | 7 | p.Gln206Arg | 0.18 | 0.02 | 43 | 0.957 |

Supplement 4: Lists of the genes used in filtration

| Filter | List of genes |
| --- | --- |
| Filter chain including genes for ADHD  (Reference: hg19) created in May 28 2018 05:29 PM) | LRRC7, SEMA3A, STIP1, C9orf62, FERMT3, TRPT1, PARD3B, DPP6, TRPA1, FRMD1, DNMT3B, MAPRE1, EFCAB8, BAALC, CPLX2, GPR139, EMP2, CEP112, BMPR1B, UGT1A8, UGT1A1, UGT1A9, UGT1A10, PBX2P1, SLC9A9, ELOVL6, TMX3, ARSB, GRIK4, NCL, UNCX, MICALL2, RARB, RUNX1T1, ADAM12, GVINP2, OR2AG2, SOX5, BCL2L1, TPX2, MYLK2, ITGA1, PTCD3, ST6GAL1, RASGRF2, MARK2, FGF14, AK8, MOBP, MAP1B, UNC5B, MAN2A2, ASTN2, CSMD2, DNM1, PPM1F, TOP3B, ITGAE, NT5DC3, CRYGC, MMP24, ITGA11, GPC6, MYT1L, REEP5, TLL2, CREB5, ATP2C2, TFEB, CDH13, SUPT3H, SORCS2, DST, ACOXL, ZNF831, NUAK1, MSH4, DLG2, CHRNB4, FAM208A, ARHGEF3, PTP4A1P4, NBPF22P, BCL11A, GRM5, TGFB2, EREG, HAS3, ZNF544, ZNF385D, NAPRT1, EIF3M, CLYBL, DOCK10, ADAMTS2, MAP3K7, TRPS1, TYRP1, SLC35F4, SULF2, MTA3, NCKAP5, DPP10, FHIT, FOXP1, NAV2, ARNT2, FBXL16, SV2C, MLIP, CSRNP3, RANBP2, DUSP6, POC1B, MEF2C, SEMA6D, ST3GAL3, DENND5B, NTM, CSMD1, KRT18P42, TEPP, ZNF319, CPNE4, MICAL2, GOT2P1, H3F3A, ACBD3, ZMAT4, SPINK2, PHLPP1, UFM1, PCDH20, MLLT1, FAM155A, LIG4, BMP7, CDH6, TTLL8, PCNPP2, C9orf47, EDN1, HIVEP1, ST18, ETV3, RGL1, FSTL4, RPS26P6, YWHAZ, PTPRD, ST13P11, KIRREL3, PAWR, PDX1, RBFOX1, PKD1L3, PKD1L2, ATP8B1, RPS3AP1, LINC00320, ESRRB, PIWIL4, MTUS2, KIF6, SLC6A6P1, SHFM1, CHMP7, TSHZ2, TRUB1, TAF9BP2, ABLIM1, NLGN1, ADCY2, RXRG, ASCC2, SUMO1P1, BCAS1, SLC4A10, PTPRG, SLC35F1, PBX4, ACTR3B, CALD1, LMOD2, FOXN3, LRRTM4, CUX1, NRL, ARHGEF12, OTOL1, DMRT2, GRIK1, MBOAT1, MEIS2, ZNF805, SPATA13, NOS1, FAM189A1, IL16, LPL, GUCY1A2, SLCO3A1, ZNF423, CHCHD2P9, TCF15, MAD1L1, GRIN2A, CLEC17A, CACNB2, PALB2, MPP6, MTCO3P1, TCF4, GLT8D1, ITIH3, NTRK3, ANKS1B, MIR137HG, IL1R1, C10orf32, ASMT, AS3MT, NEURL1B, SLC35F2, HINT1, PPP2R2B, SYNE1, KIF21B, HDAC4, DPCR1, HLA-DMB, HLA-DMA, ANK3, CACNA1C, ZNRD1, RERE, ZNF615, FTSJ2, C2orf82, ZMIZ1, ANO5, SLC17A6, ADRA1D, FOXN2, PPP1R21, PRPF3, ZNF804A, KIF5C, TRIM26, CNNM2, NT5C2 |
| Filter chain including genes for Autism.  (Reference: hg19) created in Jun 21 2018 01:17 AM) | ZBED3, TAS2R1, NAALADL2, OR2M4, OR2T33, SGSM2, PHB, MSNP1, TF, TFP1, CUEDC2, TRIM33, AMPD1, NEDD1, CSDE1, BCAS2, TUBB3, DEF8, DDAH1, IMPA2, FBXW12, RHOJ, LRRC20, PTH2R, DCLK2, CCR6, MIR146B, RIC8B, CBFA2T3, CTU2, ZNF365, PARD3B, GPR31, TAF1C, EXT1, HLA-A, MIR137HG, ZKSCAN3, HLA-DQA1, OR2N1P, OR2J2, HLA-DRB9, HLA-DRB5, NOTCH4, OR5V1, GRM3, FURIN, VRK2, HLA-DRB1, BANK1, SLC39A8, LUZP2, ACTR5, PPP1R16B, ZSWIM6, BRD2, DDR1, FOXP1, HLA-DOA, SNX19, NGEF, FHIT, HLA-DQB1, MTCO3P1, HLA-B, MKL1, BTNL2, HLA-DRB6, HLA-DRA, C6orf10, PBX2, TSNARE1, APOPT1, HIST1H2BN, HIST1H2BPS2, MAD1L1, IMMP2L, HLA-S, GALNT10, SHISA9, SFTA2, IGSF9B, DPCR1, MEF2C, PPP1R13B, GRIN2A, GRAMD1B, ETF1, RGS6, HIST1H2APS4, DPYD, CHRNA3, MDC1, TUBB, CNOT1, HCG22, PPP1R2P1, C6orf15, ATP2B2, HCG27, HLA-C, GRIA1, MDK, C2orf47, HCG4, SF3B1, HLA-DMB, GIGYF2, SRPK2, CACNB2, EPHX2, PRKG1, RIMS1, STAG1, TCF20, MAN2A1, C2, OR2W2P, OR2B7P, ZNF318, F2, NT5C2, NXPH4, BTN3A2, SHMT2, BTN2A2, KDM4A, PTPRF, TMEM219, SLC38A7, DHFRP2, FGFR1, CACNA1I, PCNX, TWF2, EEF1A2, RFT1, HCG26, GUSBP2, NRGN, TCF4, CNTN4, MICA, MUC21, SATB2, HCN1, LINC00240, MYL8P, MMP16, LYPLA2P1, TMX2-CTNND1, SNAP91, CHRNA5, ZNF823, CACNA1C, SYNGAP1, ZNF592, ALPK3, ZNF204P, DGKI, HLA-DMA, PCGEM1, PRKCD, CTC-436P18.1, SRR, HLA-DQA2, RERE, MOB4, ZNF804A, HSPE1-MOB4, MPHOSPH9, BAK1, HCG4B, TRIM8, ARL3, CLCN3, EP300, HLA-U, KDM3B, BTN2A1, MACROD2, PPP2R5C, NTM, SLTM, RASGRP4, OR2J4P, OR2H4P, MYO1E, MIR135A2, CELF4, KIF12, DOCK1, EPB41L3, OTOL1, KCNJ11, SAYSD1, LOC100506124, KCNK5, LHFPL3, LMX1B, PLCB1, RPS27L, SCN11A, TRIM10, SUMO2P1, HFE, MUC22, MOG, LINC00243, DDX39BP2, PRRC2A, TNXB, VN1R10P, FGFR3P1, UBQLN1P1, ITIH3, LRRC16A, TRIM31-AS1, MSH5, MSH5-SAPCD1, ZDHHC20P1, FKBPL, PRRT1, HCG4P5, MICD, EHMT2, TRIM26, PSORS1C1, HLA-H, ATAT1, BTN3A1, ZNF192P2, HCG4P7, HLA-T, PDZK1, REG3G, SPAG16, NEU2, OR6C64P, TCF15, CLEC17A, PALB2, MPP6, GLT8D1, NTRK3, ANKS1B, IL1R1, C10orf32-AS3MT, SLC35F2, FAM155A, LIG4, HINT1, PPP2R2B, SYNE1, KIF21B, HDAC4, ANK3, ZNRD1, ZNF615, SEMA3A, FTSJ2, GRIK1, C2orf82, ZMIZ1, ANO5, ADRA1D, FOXN2, PRPF3, KIF5C, CNNM2, CSMD1, AUTS2, SLC17A6, PPP1R21 |
| Filter chain including genes for combined ADHD/Autism  (Reference: hg19) created in Jun 23 2018 11:29 PM) | LRRC7, KIF21B, TGFB2, ACOXL, PARD3B, ADCY2, ITGA1, ARSB, FSTL4, HIVEP1, HLA-DMA, SYNE1, MICALL2, CSMD1, TRPA1, DMRT2, TYRP1, FERMT3, DUSP6, GALNT4, POC1B-GALNT4, POC1B, FAM71C, ANKS1B, NUAK1, SPATA13, MTUS2, IL16, PALB2, PKD1L2, CDH13, ZNF615, DPYD, AMPD1, NGEF, BANK1, CLCN3, HCN1, PRRC2A, EHMT2, TNXB, CYP21A2, NOTCH4, BTNL2, ZNF318, RIMS1, SNAP91, AUTS2, EPHX2, TSNARE1, SNX19, NXPH4, ALPK3, FURIN, TAF1C, CTU2, CBFA2T3, EPB41L3, RASGRP4, CACNA1I |
